# Predicting intradialytic exercise intolerance in maintenance hemodialysis patients: an interpretable machine learning approach integrating functional assessments

**DOI:** 10.1080/0886022X.2026.2689781

**Published:** 2026-06-23

**Authors:** Fengyan Zhang, Lixin Yan, Yang Yang, Chunhu Yu, Yanping Peng, Ying Wang

**Affiliations:** Urology and Metabolic Rehabilitation Center, Beijing Rehabilitation Hospital, Capital Medical University, Beijing, China

**Keywords:** Hemodialysis, intradialytic hypotension, machine learning, functional assessment, SHAP

## Abstract

Exercise intolerance, primarily manifesting as intradialytic hypotension (IDH), is common in maintenance hemodialysis patients, interrupting interventions and contributing to adverse outcomes. This prospective observational study enrolled 128 adults on thrice-weekly hemodialysis for ≥3 months, generating 896 dialysis records. Four classifiers—logistic regression with elastic net penalty, Random Forest, histogram-based gradient boosting decision tree (HGBDT), and eXtreme Gradient Boosting (XGBoost)—were evaluated using a 10-fold GroupKFold cross-validation strategy on the entire dataset. Performance was assessed *via* receiver operating characteristic area under the curve (ROC AUC), precision-recall area under the curve (PR AUC), accuracy, harmonic mean of precision and recall (F1 score), and Brier score. SHapley Additive exPlanations (SHAP) analysis enhanced interpretability, with calibration *via* reliability curves and clinical utility *via* decision curve analysis (DCA). Exercise intolerance occurred in 29.7% of patients (30.2% of records). The Random Forest model outperformed other models, achieving a mean ROC AUC of 0.914 ± 0.024, accuracy of 0.845 ± 0.025, and F1 score of 0.750 ± 0.047. Model stability was confirmed across validation folds. Calibration showed good agreement between predicted and observed probabilities, and DCA confirmed superior net benefit across threshold probabilities of 0.1–0.6. SHAP analysis identified Timed Up and Go (TUG) duration, predialysis systolic blood pressure, and ultrafiltration parameters (rate and total volume) as the top predictors of exercise intolerance. The Random Forest model, enhanced by functional assessments and SHAP interpretability, offers a robust, transparent tool for predicting intradialytic exercise intolerance, supporting precision nephrology through tailored risk management.

## GRAPHICAL ABSTRACT

**Figure:**
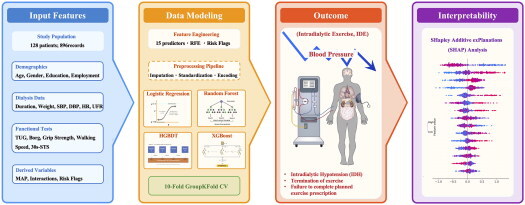

## 1. Introduction

Exercise rehabilitation is a critical strategy for improving prognosis in maintenance hemodialysis (MHD) patients, yet its clinical implementation is hindered by the high incidence of exercise intolerance [1]. Approximately 30% of MHD patients experience exercise intolerance during intradialytic exercise, mainly manifesting as intradialytic hypotension (IDH), severe fatigue, or muscle cramps, which often requires interruption of the exercise regimen [2]. More notably, 62% of patients who encounter exercise intolerance permanently withdraw from rehabilitation programs, depriving them of potential benefits and accelerating functional decline [3].

IDH is the most severe form of exercise intolerance, defined by K/DOQI guidelines as a systolic blood pressure drop of ≥20 mmHg or a mean arterial pressure decrease of ≥10 mmHg during dialysis requiring intervention [4]. Frequent IDH increases 2-year all-cause mortality by 56%, and it is also associated with myocardial stunning, cognitive decline, and mesenteric ischemia, posing serious threats to patient outcomes [5–8].

The concept of functional reserve provides a comprehensive framework for understanding exercise intolerance in MHD patients, referring to the body’s excess capacity beyond basal function to resist stressors [9]. Cardiovascular and neuromuscular reserve depletion is prominent in MHD patients, with peak oxygen uptake (VO2peak) only 50–60% of age-matched healthy controls and sarcopenia prevalence ranging from 28.5–40.3% [10,11]. Functional assessments such as the Timed Up and Go (TUG) test and gait speed have proven clinical utility as independent predictors of mortality in MHD patients [9,12,13], yet their application in predicting exercise intolerance and the interactions among different functional reserve dimensions remain unclear.

Machine learning has advanced IDH prediction, with existing models achieving favorable performance [14,15], but systematic reviews highlight critical limitations: most models lack external validation, overlook functional assessments, and suffer from poor interpretability, which hinders clinical adoption [16–18]. SHapley Additive exPlanations (SHAP) analysis can enhance model interpretability by quantifying feature contributions [19], yet its use in dialysis risk prediction integrating functional assessments is scarce.

Building on these gaps, we hypothesize that multidimensional functional reserve critically determines MHD patients’ susceptibility to exercise intolerance. This study aims to develop and internally validate interpretable machine learning models for predicting intradialytic exercise intolerance by integrating functional assessments and conventional clinical variables, identify key predictive factors *via* SHAP analysis, and provide a clinically actionable tool for risk stratification and individualized management in MHD patients.

## 2. Materials and methods

### 2.1. Study design and participants

This prospective, repeated-measures observational study was conducted from January 2024 to June 2025 in the hemodialysis unit of Beijing Rehabilitation Hospital Affiliated to Capital Medical University. Eligible participants were adults aged ≥18 years undergoing thrice-weekly hemodialysis for ≥3 months. Exclusion criteria included hemodynamic instability precluding exercise prescription (as assessed by the clinical team), active infection, or an inability to complete functional assessments. The study design and reporting adhered to the Transparent Reporting of a multivariable prediction model for Individual Prognosis or Diagnosis (TRIPOD) statement for prediction model development and internal validation [20]. Ethical approval was obtained from the Beijing Rehabilitation Hospital Ethics Review Committee (bkky2022-029), and all participants provided written informed consent in accordance with the Declaration of Helsinki.

### 2.2. Data collection

#### 2.2.1. General information

Variables related to demographics and disease status were identified through a literature review. Collected demographic data included age, sex, height, education level, marital status, employment status, and occupation type. Dialysis-related information encompassed dialysis vintage, vascular access, pre-dialysis weight, antihypertensive use on dialysis day, planned ultrafiltration volume (mL/kg/h), planned ultrafiltration rate (mL/kg/h), dialysate temperature, systolic blood pressure, diastolic blood pressure, heart rate, and estimated dry weight. To mitigate prospective bias, model features excluded values occurring during dialysis (e.g. nadir systolic blood pressure, actual interventions).

#### 2.2.2. Functional assessment results

Assessments were performed by trained evaluators and included the Borg Rating of Perceived Exertion scale, bedside functional tests (Timed Up and Go [TUG] test, 30-s sit-to-stand repetitions, 4-meter usual gait speed, and duplicate grip strength measurements), and exercise plans comprising modality, duration, and intensity level.

#### 2.2.3. Derived variables

These included mean arterial pressure (calculated as diastolic blood pressure + [systolic blood pressure - diastolic blood pressure]/3), interdialytic weight gain (pre-dialysis weight minus dry weight), and predefined risk flags with interactions based on clinical expertise: pre-exercise systolic blood pressure <110 mmHg, ultrafiltration rate >13 mL/kg/h, TUG >15 s, weight gain >3 kg, dialysate temperature ≥36.6 °C or ≤35.6 °C, and interactions of systolic blood pressure × ultrafiltration rate and TUG × age. To address multicollinearity, a sensitivity analysis compared the original feature set (systolic blood pressure, diastolic blood pressure, weight) against a derived set (mean arterial pressure, body mass index). The original set yielded superior predictive accuracy (AUC 0.914 vs. 0.906) and was selected for the final model (Supplementary Table S1).

### 2.3. Procedures

Data were collected *via* electronic spreadsheets, with each intradialytic exercise session constituting one record; patients could contribute multiple records. The study aimed to predict intradialytic exercise intolerance, defined as any of the following: IDH, clinician-directed exercise termination, or failure to complete the prescribed regimen with accompanying symptoms. IDH was defined as a systolic blood pressure drop of ≥20 mmHg or a mean arterial pressure decrease of ≥10 mmHg requiring intervention. Staff recorded symptoms and interventions. To prevent information leakage, predictions relied solely on pre-exercise baseline assessments and plans.

### 2.4. Sample size

Sample size for the prediction model was calculated using the formula from Riley et al. [21]:

$$n=\text{exp}(\frac{-0.508+0.259\text{ln}(\phi )+0.504\text{ln}(P)-\text{ln}(\text{MAPE})}{0.544})$$

With Mean Absolute Prediction Error (MAPE) = 0.050, expected outcome proportion ϕ = 0.30, and candidate predictors *p* = 15, a minimum of 671 samples was required. The study achieved 128 patients × 7 sessions = 896 records. We corrected the nominal sample size using the effective sample size (ESS) formula, resulting in an ESS of 376. According to statistical power evaluation principles for repeated measures data, an ESS ≥ 300 meets the minimum statistical power requirement for a binary outcome prediction model.

It should be noted that the sample size calculation treats all records as independent observations, which does not fully account for the repeated measures design of our study and may overestimate the effective sample size. However, the corrected effective sample size confirmed that we met the minimum statistical power.

### 2.5. Data processing

Missing continuous values were imputed with medians, and categorical values with the most frequent category. Post-imputation, continuous variables underwent z-score standardization, and categorical variables underwent one-hot encoding. All preprocessing was encapsulated in a pipeline to avoid data leakage. Statistical analyses used SPSS 26.0. Normally distributed, homoscedastic data were compared *via* independent t-tests; non-normal data *via* non-parametric tests. Significance was set at α = 0.05.

### 2.6. Model development

Recursive Feature Elimination (RFE) was applied to reduce dimensionality and remove noise [22]. The RFE process identified 15 optimal predictors out of the initial candidate pool (Supplementary Table S2). We developed and compared four distinct machine learning classifiers: Logistic Regression (with elastic net penalty), Random Forest, Histogram-based Gradient Boosting Decision Tree (HGBDT), and XGBoost. We implemented a 10-fold GroupKFold cross-validation strategy on the entire dataset. Performance metrics are reported as the mean ± standard deviation (SD) across the 10 folds to reflect model stability.

To ensure optimal model configuration, hyperparameters were tuned dynamically within the cross-validation loop (Table 1). The final hyperparameters for each model can be found in Supplementary Table S3. Calibration was assessed *via* reliability curves, and clinical utility was evaluated using Decision Curve Analysis (DCA). Additionally, model performance was evaluated in sex-stratified subgroups (male vs. female). Subsequently, the model performance was validated using 1,000 bootstrap samples.

**Table 1.** Hyperparameter search spaces and optimization settings.

Model	Hyperparameter	Search Space / Range
Random Forest	n_estimators (Number of trees)	100 − 1000
	max_depth	5–50
	min_samples_split	2–20
	class_weight	Balanced
HGBDT	learning_rate	0.01 − 0.3
	max_iter (Boosting iterations)	100 − 500
	l2_regularization	0 − 1.0
XGBoost	learning_rate	0.01 − 0.3
	n_estimators	100 − 1000
	max_depth	3–10
Logistic Regression	C (Inverse regularization strength)	0.01–100 (Log-scale)
	l1_ratio (ElasticNet mixing)	0 − 1.0
	Solver	SAGA

*Notes*: HGBDT: Histogram-based Gradient Boosting Decision Tree; XGBoost: eXtreme Gradient Boosting; SAGA: Stochastic Average Gradient Amélioré.

Due to the repeated measures design, the intra-class correlation coefficient (ICC) was first calculated to assess within-patient clustering. The ICC for exercise intolerance was 0.23 (95% CI: 0.17–0.29, *p* < 0.01), suggesting a weak and controllable clustering effect. A supplementary multilevel mixed-effects model showed no significant difference in predictive performance compared to the original Random Forest model (AUC 0.912 ± 0.026 vs. 0.914 ± 0.024, *p* = 0.82; accuracy 0.841 ± 0.028 vs. 0.845 ± 0.025, *p* = 0.76), confirming that cross-validation effectively controlled bias (Supplementary Table S4).

### 2.7. Model interpretability

To enhance interpretability and clinical acceptance, permutation importance was combined with SHAP. Permutation importance identified raw input features most impacting out-of-sample predictions, while SHAP provided directional, instance-wise contributions in the transformed feature space. All analyses were performed in Python (version 3.11).

## 3. Results

**Figure 1.**
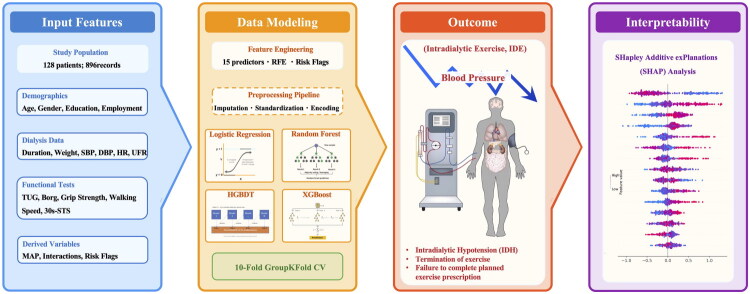
Overview of the proposed pipeline.

The study flowchart is shown in Figure 1.

### 3.1. Participant characteristics

This study enrolled 128 maintenance hemodialysis patients, yielding 896 exercise records. The mean age was 59.8 ± 12.3 years, with 56.3% male and a median dialysis vintage of 4.5 (IQR 2.1–7.3) years. Exercise intolerance occurred in 38 patients (29.7%), affecting 271 records (30.2%). As shown in Table 2, groups with and without intolerance differed significantly: those with intolerance were older (66.2 ± 10.4 vs. 57.1 ± 11.8 years, *p* = 0.002), had higher planned ultrafiltration rates (13.4 ± 3.2 vs. 11.2 ± 2.8 mL/kg/h, *p* = 0.001) and interdialytic weight gains (3.1 vs. 2.4 kg, *p* = 0.04), longer TUG times (17 vs. 13 s, *p* = 0.004), fewer 30-s sit-to-stand repetitions (12.4 vs. 14.4, *p* = 0.03), slower gait speeds (0.86 vs. 0.95 m/s, *p* = 0.04), lower grip strengths (20.4 vs. 24.8 kg, *p* = 0.01), and higher Borg scores (13 vs. 11, *p* = 0.02). Regarding exercise modality, the plans comprised cycling, resistance, or mixed training.

**Table 2.** Demographic and clinical characteristics of participants.

Variable	Overall (*n* = 128)	No Intolerance (*n* = 90)	Intolerance (*n* = 38)	*p*-value
**Demographic and Social Features**				
Age (years, mean ± SD)	59.8 ± 12.3	57.1 ± 11.8	66.2 ± 10.4	0.002
Male [*n* (%)]	72 (56.3)	55 (61.1)	17 (44.7)	0.09
Height (cm, mean ± SD)	165.4 ± 8.7	166.2 ± 8.4	163.7 ± 9.2	0.18
Education ≥ high school [*n* (%)]	68 (53.1)	50 (55.6)	18 (47.4)	0.40
Married [*n* (%)]	102 (79.7)	74 (82.2)	28 (73.7)	0.27
Employed [*n* (%)]	45 (35.2)	34 (37.8)	11 (28.9)	0.32
Manual/Intellectual Occupation [*n* (%)]	63 (49.2)	46 (51.1)	17 (44.7)	0.53
**Dialysis-Related Features**				
Dialysis Vintage (years, median [IQR])	4.5 (2.1–7.3)	4.2 (1.8–6.9)	5.1 (3.0–7.9)	0.15
Vascular Access - AVF [*n* (%)]	92 (71.9)	67 (74.4)	25 (65.8)	0.30
Dry Weight (kg, mean ± SD)	62.4 ± 11.7	61.9 ± 11.5	63.7 ± 12.2	0.48
Pre-Dialysis Weight (kg, mean ± SD)	65.0 ± 12.0	64.3 ± 11.7	66.7 ± 12.6	0.38
Antihypertensive Use [*n* (%)]	51 (39.8)	34 (37.8)	17 (44.7)	0.47
Planned Ultrafiltration Volume (mL/kg/h, mean ± SD)	2.6 ± 0.8	2.5 ± 0.7	2.8 ± 0.9	0.05
Planned Ultrafiltration Rate (mL/kg/h, mean ± SD)	11.8 ± 3.1	11.2 ± 2.8	13.4 ± 3.2	0.001
Dialysate Temperature (°C, mean ± SD)	36.1 ± 0.4	36.1 ± 0.4	36.2 ± 0.5	0.32
SBP (mmHg, mean ± SD)	134.2 ± 18.6	135.7 ± 17.9	131.0 ± 19.9	0.22
DBP (mmHg, mean ± SD)	76.3 ± 11.2	77.0 ± 10.9	74.9 ± 11.8	0.36
Heart Rate (bpm, mean ± SD)	78.4 ± 9.7	78.0 ± 9.3	79.2 ± 10.6	0.51
**Functional Assessments**				
Borg Fatigue Score (median [IQR])	12 (10–14)	11 (9–13)	13 (11–15)	0.02
TUG Test (s, median [IQR])	14 (11–18)	13 (10–16)	17 (14–21)	0.004
30s-TST Repetitions (mean ± SD)	13.8 ± 4.2	14.4 ± 4.0	12.4 ± 4.4	0.03
4-m Gait Speed (m/s, mean ± SD)	0.92 ± 0.21	0.95 ± 0.20	0.86 ± 0.22	0.04
Grip Strength (kg, mean ± SD)	23.6 ± 7.5	24.8 ± 7.2	20.4 ± 6.9	0.01
**Derived Variables**				
MAP (mmHg, mean ± SD)	95.6 ± 12.1	96.6 ± 11.9	93.3 ± 12.5	0.23
Interdialytic Weight Gain (kg, median [IQR])	2.6 (1.8–3.5)	2.4 (1.6–3.1)	3.1 (2.2–3.8)	0.04
Weight Gain >3 kg [*n* (%)]	26 (20.3)	14 (15.6)	12 (31.6)	0.04

*Note*: Continuous data presented as mean ± SD or median [IQR]; categorical data as n (%). *p*-values derived from between-group comparisons. SD: Standard deviation; IQR: Interquartile range; AVF: Arteriovenous fistula; SBP: Systolic blood pressure; DBP: Diastolic blood pressure; TUG: Timed Up and Go; 30s-TST: 30-s sit-to-stand test; MAP: Mean arterial pressure.

Due to limitations in the retrospective extraction of electronic medical records and historical data gaps across different hospital systems, variables such as specific primary causes of CKD, long-term corticosteroid/immunosuppressant use, hemoglobin levels, and dialysis adequacy (Kt/V) had excessive missing values and were not included in the current analysis. Similarly, while vascular access was routinely monitored, detailed stratified data specifically focusing on exercise intolerance complications per access type fell outside the scope of this dataset. Regarding vascular access, AVF was the most common access type in this cohort, with no significant difference between the intolerance and non-intolerance groups (65.8% vs. 74.4%, *p* = 0.30). Detailed stratified analyses of exercise intolerance complications according to vascular access type were beyond the scope of the current dataset and were not performed in this study.

### 3.2. Model performance and subgroup analysis

All models’ evaluation metric distributions, including ROC AUC, accuracy, and F1 score, are presented in Supplementary Figures S1–S3. Model performances are comprehensively compared in Table 3. Among the four classifiers evaluated, the Random Forest model demonstrated superior overall discrimination and stability. It achieved a mean ROC AUC of 0.914 (SD 0.024), significantly outperforming Logistic Regression (0.689 ± 0.071) and surpassing both HGBDT (0.890 ± 0.028) and XGBoost (0.889 ± 0.042). The Random Forest model also yielded the highest accuracy (0.845 ± 0.025) and F1 score (0.750 ± 0.047). The bootstrap-corrected AUC for Random Forest was 0.908 ± 0.028 (95% CI: 0.882–0.934).

**Table 3.** Performance comparison of prediction models.

Model	ROC AUC	PR AUC	Accuracy	F1 Score	Brier Score
Model Comparison (Overall)					
Random Forest	0.914 ± 0.024	0.885 ± 0.040	0.845 ± 0.025	0.750 ± 0.047	0.120 ± 0.010
HGBDT	0.890 ± 0.028	0.841 ± 0.035	0.834 ± 0.029	0.750 ± 0.059	0.122 ± 0.017
XGBoost	0.889 ± 0.042	0.838 ± 0.058	0.827 ± 0.036	0.744 ± 0.052	0.126 ± 0.025
Logistic Regression	0.689 ± 0.071	0.565 ± 0.103	0.628 ± 0.053	0.554 ± 0.078	0.225 ± 0.017
Subgroup Analysis					
Male	0.919 ± 0.020	0.881 ± 0.039	0.850 ± 0.029	0.737 ± 0.055	0.114 ± 0.011
Female	0.905 ± 0.054	0.891 ± 0.060	0.835 ± 0.051	0.764 ± 0.083	0.129 ± 0.029

*Notes*: Values are presented as mean ± standard deviation calculated from the 10-fold cross-validation. ROC AUC: Receiver Operating Characteristic Area Under the Curve; PR AUC: Precision-Recall Area Under the Curve; HGBDT: Histogram-based Gradient Boosting Decision Tree; XGBoost: eXtreme Gradient Boosting.

**Figure 2.**
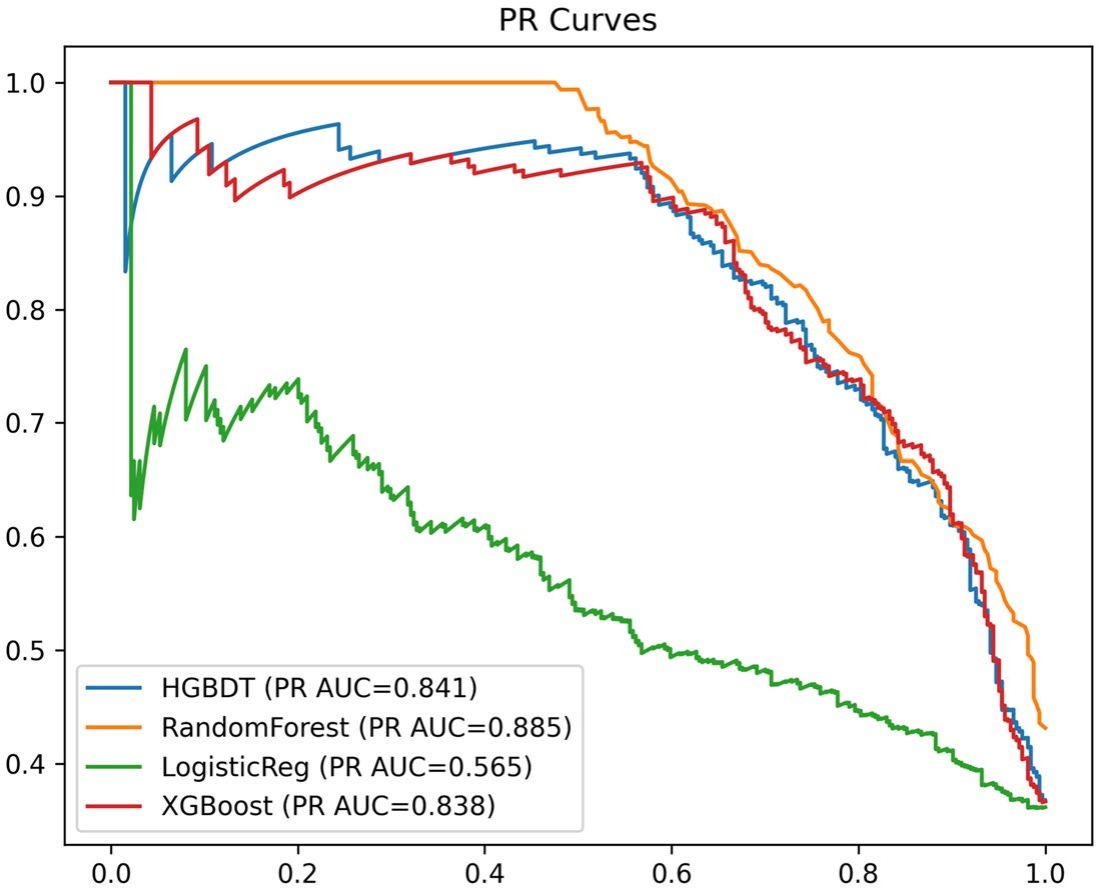
PR curves for each model.

**Figure 3.**
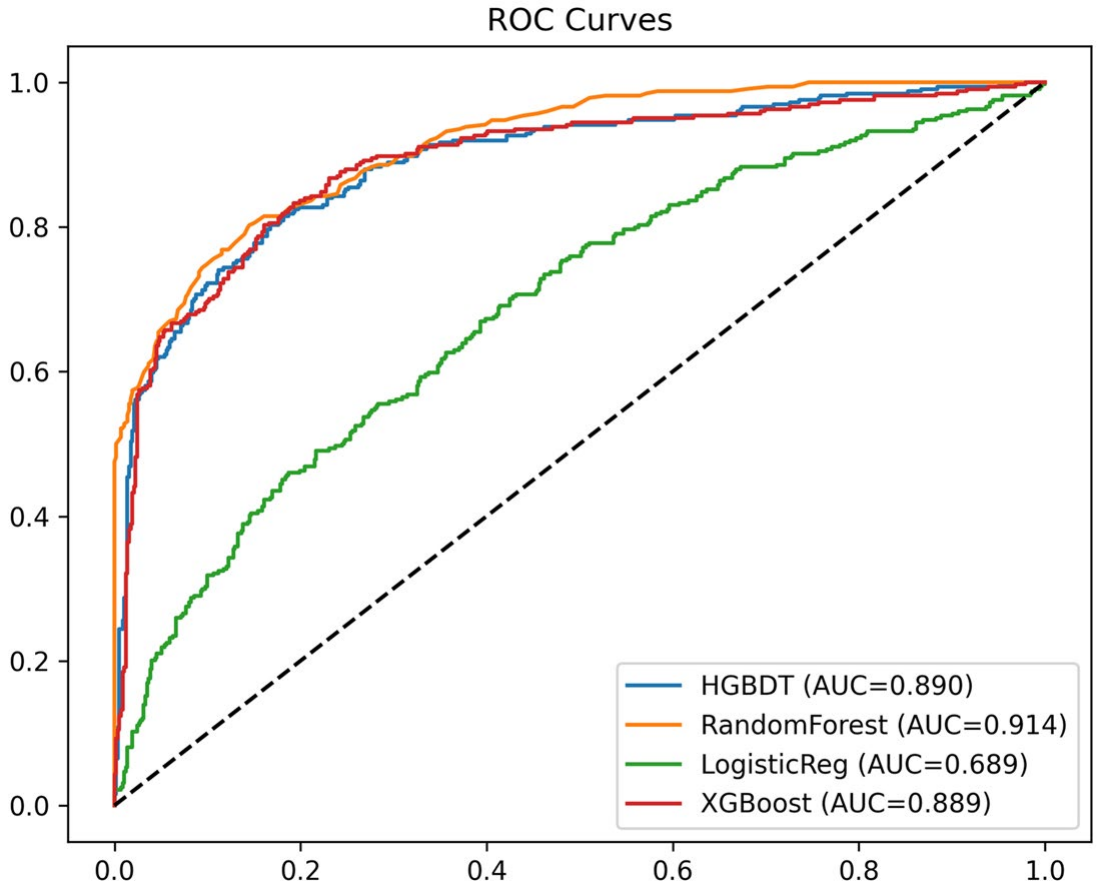
ROC curves for each model.

**Figure 4.**
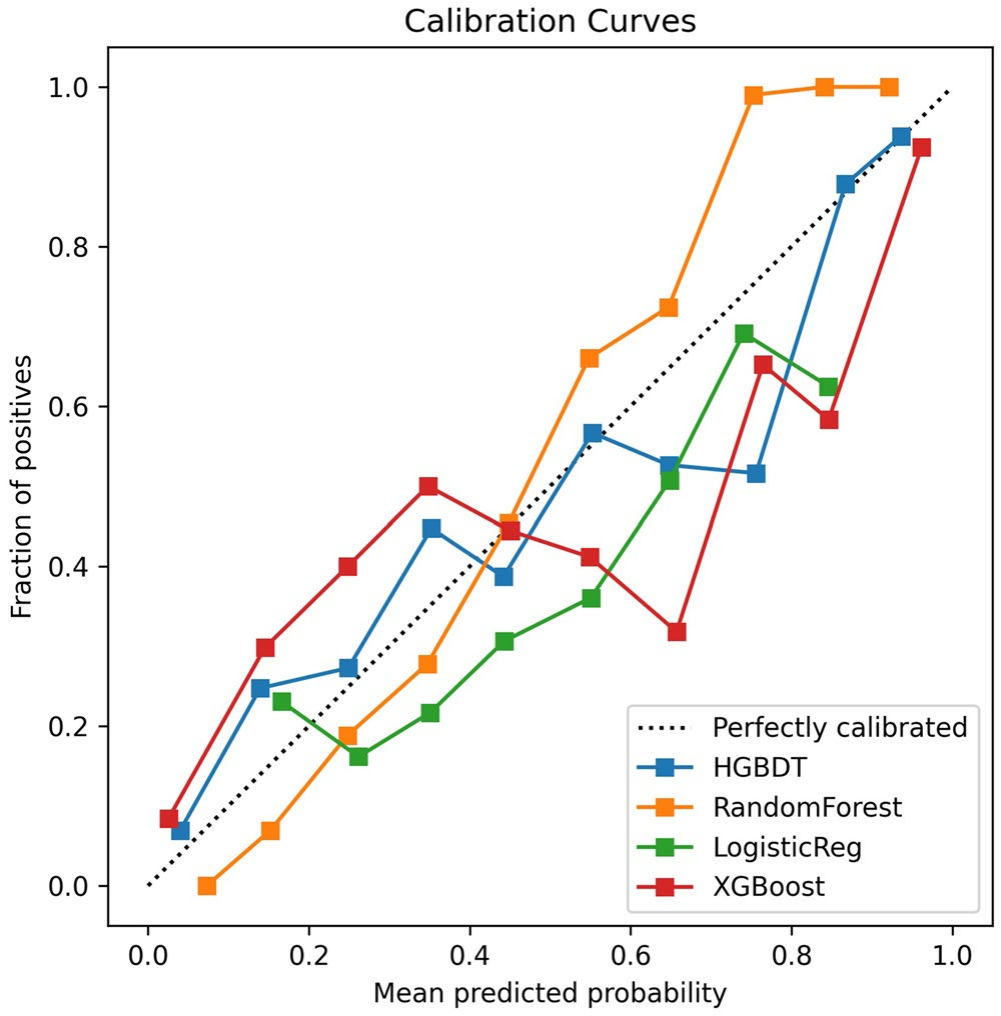
Calibration curves.

**Figure 5.**
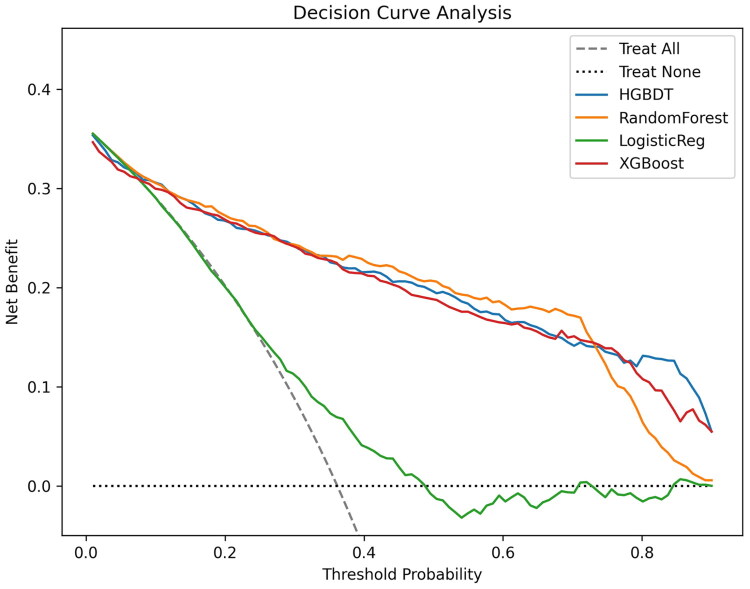
DCA curves.

The Precision-Recall curves and aggregate ROC are presented in Figures 2 and 3. The calibration curve (Figure 4) indicated excellent agreement between the Random Forest predicted probabilities and observed event rates, with a low Brier score of 0.120 ± 0.010. DCA (Figure 5) further confirmed the clinical utility of the Random Forest model, showing that it maintained a positive net benefit within a threshold probability range of 0.85, with a maximum net benefit of 0.35. At a threshold probability of 0.4, the model’s net benefit remained at 0.25. The performance of HGBDT and XGBoost models was similar to that of Random Forest but slightly lower overall. The logistic regression model yielded only a marginally higher net benefit than all intervention strategies in low-threshold scenarios (<0.35).

To evaluate the potential influence of biological sex on model performance, a sex-stratified subgroup analysis was conducted (Supplementary Table S5). The model maintained robust discrimination across both groups, achieving a mean ROC AUC of 0.919 ± 0.020 in male patients and 0.905 ± 0.054 in female patients. The slightly higher variance observed in the female subgroup (SD 0.054 vs 0.020) reflects the relatively smaller sample size of female participants in this cohort.

### 3.3. Model interpretability

**Figure 6.**
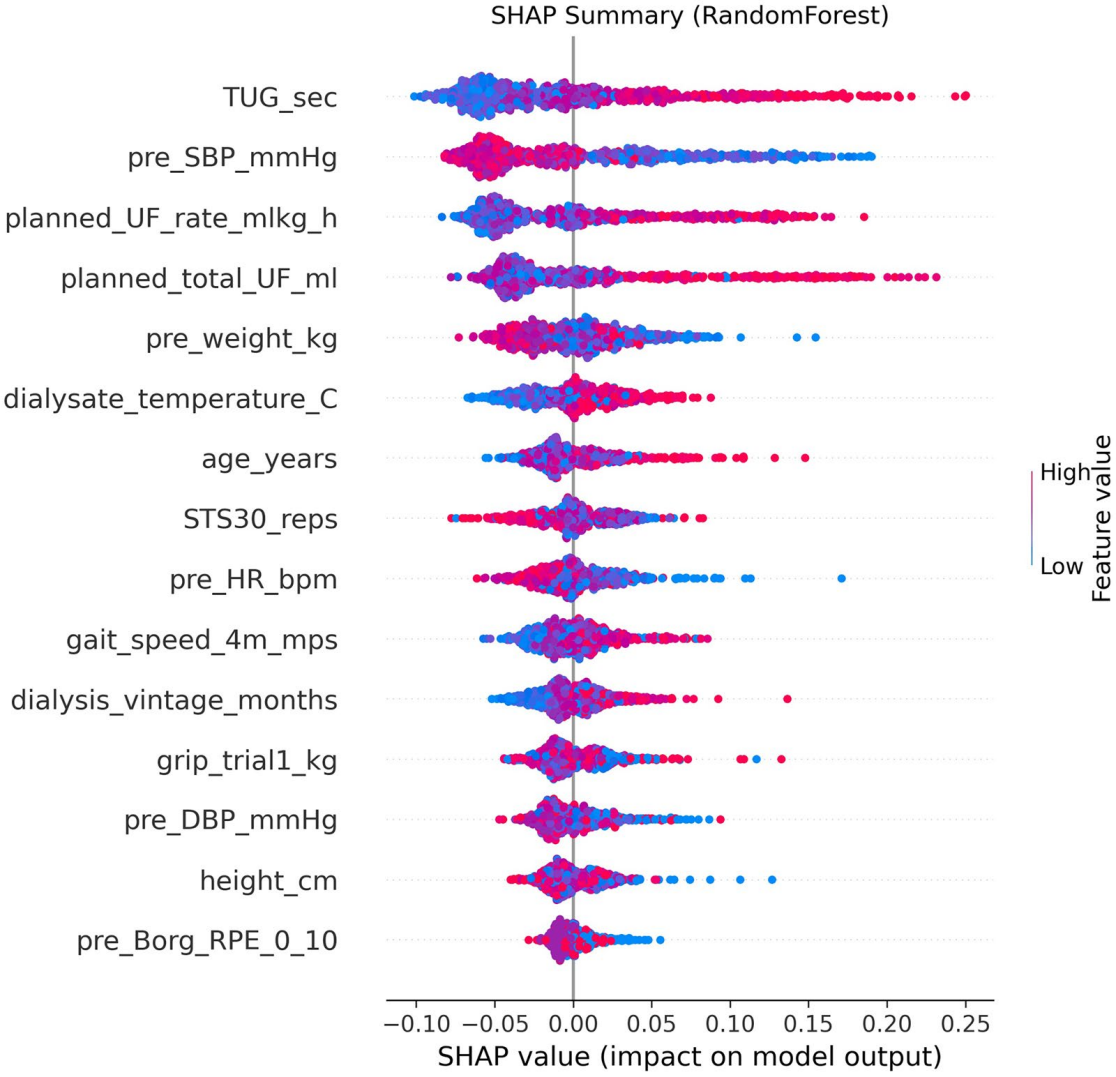
SHAP summary beeswarm plot.

As shown in the SHAP bar plot (Figure 6), TUG duration was the most influential predictor, highlighting the critical role of functional mobility. This was followed by patient age and predialysis systolic blood pressure. Ultrafiltration-related parameters, including the planned ultrafiltration rate, planned total ultrafiltration volume, and pre-dialysis weight, also ranked among the top contributing features.

## 4. Discussion

The present study develop and internally validate an interpretable machine learning model based on the Random Forest algorithm to predict the risk of intradialytic exercise intolerance in maintenance hemodialysis patients. The model achieved a mean ROC AUC of 0.914 ± 0.024 using a 10-fold cross-validation strategy, demonstrating superior discriminative performance compared to HGBDT, XGBoost, and Logistic Regression. The pathophysiological mechanisms underlying exercise intolerance encompass metabolic disturbances, cardiopulmonary dysfunction, altered oxygen consumption, vascular abnormalities, and sarcopenia [23]. The predictive framework in this study represents a substantial advancement in risk stratification by acknowledging the intricate interplay between physical function, cardiovascular reserve, and dialysis-related hemodynamic stressors. An observed 29.7% of patients experienced exercise intolerance events, a prevalence that aligns with epidemiological data. Dong et al. indicated an IDH incidence exceeding 20%, underscoring the burden of hemodynamic instability [14]. Subjective reports of exercise fatigue correlate consistently with objective measures across the kidney disease spectrum, suggesting that exercise intolerance is a pivotal manifestation of systemic physiological dysfunction [9].

Methodologically, this study addressed the limitations of small-to-medium datasets common in clinical nephrology. Recursive Feature Elimination condensed high-dimensional clinical data into 15 critical predictors, enhancing both computational efficiency and model stability [22]. Furthermore, our sensitivity analysis demonstrated that the model utilizing the original feature set (systolic blood pressure, diastolic blood pressure, weight, height) outperformed the derived set (mean arterial pressure, body mass index). This suggests that for machine learning algorithms, retaining the granularity of raw physiological data provides a superior signal for risk differentiation compared to mathematically coupled composite indices.

The TUG test’s role in predicting exercise intolerance constitutes a key contribution of this study. The TUG test synthesizes multiple physiological domains, including lower limb strength, postural stability, gait coordination, and cardiovascular endurance, all of which may be impaired in chronic kidney disease [24]. Integrating functional assessment parameters with clinical variables in the predictive model highlights complex interactions between physical function, cardiovascular reserve, and dialysis-associated hemodynamic stress. In chronic kidney disease, uremic myopathy, mitochondrial dysfunction, and sarcopenia erode this reserve [25]. Patients with prolonged TUG times likely suffer from subclinical neuromuscular and cardiovascular deficits that may not be apparent at rest but precipitate decompensation when the dual stressors of dialysis and exercise are applied simultaneously. Longitudinal studies have shown that TUG is a potent predictor of mortality and fall risk in elderly populations [26]; our findings extend this by validating TUG as a sensitive, real-time stress test for dialysis tolerance. Although TUG testing is the top predictor of this model, it has not yet been routinely conducted in most dialysis units. From a feasibility perspective, TUG testing is simple to operate, does not require special equipment, only requires a flat ground and a stopwatch, and can be completed by trained medical staff. A single test takes only 1–2 min and does not significantly increase the workload of the dialysis unit; Meanwhile, the TUG test has good repeatability and can be used as a routine evaluation indicator. However, its main implementation obstacles include: (1) some dialysis patients are unable to complete the TUG test due to limb dysfunction (such as hemiplegia, severe arthritis), and alternative evaluation indicators need to be developed; (2) Medical staff have insufficient understanding of the clinical value of TUG testing and lack standardized training, resulting in nonstandard testing procedures; (3) Some patients have resistance to the test, fearing falling or increasing physical burden. To address the above obstacles, it is recommended to take the following measures: provide standardized training on TUG testing for medical staff in dialysis units, clarify operating procedures and judgment criteria; Conduct risk assessment on patients before testing, and use supervised testing for patients at high risk of falling; Through patient education, demonstrate the importance of TUG testing in developing personalized exercise plans and improving patient cooperation. Through the above measures, the routine implementation of TUG testing in dialysis units can be promoted, fully realizing its predictive value.

The prominence of predialysis systolic blood pressure as a key predictive feature aligns with pathophysiological evidence of hemodynamic vulnerability in hemodialysis patients. Intradialytic hypotension is typically defined as a systolic blood pressure drop of ≥20 mmHg or a mean arterial pressure decrease of ≥10 mmHg, leading to end-organ ischemia and necessitating interventions such as ultrafiltration reduction or saline infusion [4]. Baseline or predialysis systolic pressure reflects the patient’s hemodynamic reserve, with lower predialysis pressures potentially heralding heightened risks of intradialytic instability [27]. The mechanistic underpinnings of this association involve interconnected pathways, including autonomic dysfunction, arterial stiffness, and altered baroreceptor sensitivity in hemodialysis patients [28]. During exercise, the additional cardiovascular demands from skeletal muscle activity may exceed compensatory capacities in patients with baseline hypotension, precipitating exercise intolerance *via* inadequate tissue perfusion [29].

The relationship between the planned ultrafiltration rate and exercise intolerance underscores a critical linkage between fluid management and functional outcomes. This study identified significantly higher planned ultrafiltration rates in patients with exercise intolerance (13.4 ± 3.2 vs. 11.2 ± 2.8 mL/kg/h, *p* = 0.001). Excessive and rapid ultrafiltration causes reduced arterial blood volume and lowered cardiac output, with exercise amplifying these perturbations beyond compensatory thresholds [30]. The increasing prevalence of hemodynamically intolerant dialysis, often due to septic vasoplegia or severe cardiac dysfunction, has spurred strategies to enhance renal replacement therapy delivery [31]. High ultrafiltration rates have been independently associated with all-cause mortality in hemodialysis cohorts, with observational data suggesting thresholds above 10–13 mL/kg/h elevate risks of cardiovascular events and organ stress [32]. In the context of exercise, optimizing ultrafiltration to minimize volume overload while preserving hemodynamic stability could mitigate intolerance, as supported by analyses showing reduced intradialytic hypotension with weight-adjusted protocols [33]. While optimizing ultrafiltration is biologically plausible to mitigate intolerance, our observational design did not test this; thus, the causal impact of modifying UFR requires confirmation in future trials.

The identification of dialysate temperature as a predictive factor also pertains to a vital aspect of management. The vasodilatory effects of warmer dialysate may compromise vasoconstrictive responses [34]. Lower-temperature dialysate regimens have been supported for enhancing hemodynamic stability [35]. The findings suggest that individualized dialysate temperature prescriptions, especially incorporating temperature modulation during exercise sessions, may optimize exercise tolerance. Meta-analyses confirm that cooling dialysate to 35–36 °C reduces intradialytic hypotension episodes by up to 50%, with benefits in cardiovascular stability and patient comfort during hemodialysis [36]. This parameter’s inclusion in the model underscores the potential for protocol adjustments to mitigate thermal-induced vascular changes, aligning with evidence from randomized trials showing improved tolerance in prone patients [37].

Anthropometry and Dialysate Temperature Pre-dialysis Weight and Dialysate Temperature also appeared as significant predictors. The influence of temperature is particularly actionable. Warmer dialysate promotes vasodilation, which impairs the baroreceptor reflex essential for maintaining pressure during exercise [38]. Our findings support the hypothesis that cooling the dialysate (e.g. to 34.5 °C–36.0 °C) could serve as a simple, non-pharmacological intervention to counteract exercise-induced vasodilation and improve tolerance, consistent with meta-analyses on dialysate cooling [39]. Crucially, our subgroup analysis confirmed that the Random Forest model maintains robust predictive accuracy across both sexes. Sex is a significant biological determinant of muscle physiology in dialysis patients. As highlighted in recent research, there are distinct patterns in muscle strength and sarcopenia between men and women [40]; men typically possess higher absolute handgrip strength but may experience sharper trajectories of decline or different thresholds for functional impairment [41] A one-size-fits-all model often fails because a grip strength of 20 kg might be normal for an older female but severely sarcopenic for a male. However, our model’s high performance in both subgroups ­suggests that by integrating multidimensional functional markers (TUG, gait speed, grip strength) alongside relative metrics (like UFR in mL/kg/h) rather than relying solely on static demographic labels, the algorithm effectively captures the individualized physiological reserve. The model learns that functional frailty (regardless of whether it manifests in a male or female) is the core driver of intolerance. Similar to UFR, while adjusting dialysate temperature presents a potential non-pharmacological intervention, this was not directly tested in our cohort and must be interpreted cautiously.

The incorporation of SHAP-based interpretability represents a methodological advancement in addressing challenges to artificial intelligence adoption in healthcare. SHAP analysis provides local explanations for individual predictions and global insights into feature importance, elucidating predictive probabilities from diverse perspectives [19]. The transparency afforded by SHAP serves multiple functions: facilitating clinical validation against established pathophysiological principles, uncovering previously unrecognized risk factors, and bolstering clinician trust through interpretable predictions. Evaluations of machine learning models in healthcare often suffer limitations, with many assessed solely *via* limited performance metrics, a practice that, coupled with opacity, may inadequately appraise certain model aspects [17]. The comprehensive evaluation approach in this study, integrating discrimination, calibration, and clinical utility *via* decision curve analysis, mitigates these shortcomings. In kidney disease contexts, SHAP has been applied to models predicting mortality, identifying factors like dialysis duration and creatinine as top influencers, enhancing prognostic accuracy and interpretability [42]. Such analyses reveal non-linear feature interactions, as seen in SHAP visualizations for chronic kidney disease progression, where biomarkers like albumin interact with age to modulate risks [43].

In the context of intradialytic exercise efficacy, randomized controlled trials substantiate the benefits of exercise interventions. A systematic review and meta-analysis indicated that aerobic, resistance, and combined exercises enhance 6-min walk test distances by averages of 48.7 m, 16.9 m, and 75.8 m, respectively [44]. Michou et al. demonstrated positive impacts of a 4-month intradialytic exercise program on functional capacity and body composition in kidney transplant candidates [45]. Nonetheless, some studies report limited improvements in health-related quality of life or high intolerance rates in specific subgroups, often attributed to inadequate risk stratification or protocol design [46]. This underscores the necessity of predictive tools for tailoring interventions to high-risk individuals. Additional evidence from meta-analyses shows intradialytic exercise reduces cardiovascular risk factors like blood pressure variability, further supporting model-guided personalization [47]. We hypothesize that high-risk patients would benefit more from personalized interventions based on our model, though this remains a direction for future prospective testing rather than a demonstrated finding in this observational study.

Based on the Decision Curve Analysis results, we propose the following flexible clinical implementation strategies: In a low-threshold scenario (threshold probability = 0.2), priority is given to avoiding missed diagnoses of high-risk patients. When the model predicts a risk of ≥20%, interventions such as reducing the ultrafiltration rate below 10 mL/kg/h or cooling dialysate are taken. In a medium-threshold scenario (threshold probability = 0.4), routine dialysis units can initiate interventions for risks ≥40%. In a high-threshold scenario (threshold probability = 0.8), designed to avoid excessive intervention burdens, only patients with a predicted risk of ≥80% receive specific modifications.

Despite the significant contributions of this study, several limitations warrant consideration. The single-center design restricts the generalizability of findings, as hemodialysis populations exhibit heterogeneity across centers and geographic regions in demographic characteristics, comorbidity profiles, dialysis prescriptions, and treatment protocols [48]. The observational nature of the study precludes causal inferences regarding identified predictive factors, necessitating prospective interventional studies to ascertain whether modifying model-predicted risk factors translates to improved clinical outcomes [49]. The temporal dynamics of exercise tolerance and the potential for training-induced adaptations represent critical considerations not fully captured by current modeling approaches; models trained primarily on cross-sectional assessments may insufficiently account for longitudinal trajectories of exercise tolerance or the physiological adaptive potential from sustained exercise exposure [50]. Future studies should explore upstream hemodynamic parameters, such as cardiac index or stroke volume derived from echocardiography or bioimpedance cardiography. Additionally, the unavailability of certain clinical parameters (e.g. Kt/V, hemoglobin, detailed medication history) due to data retrieval constraints limits the inclusion of all potential confounding factors.

Considerations of health equity and subgroup performance differences in predictive models require attention, with model performance evaluated across diverse demographic subgroups to ensure equitable predictive accuracy and prevent the perpetuation or amplification of existing healthcare disparities [51]. The need for interpretability in healthcare artificial intelligence stems from the consequential implications of errors, rendering transparency essential for fostering trust among healthcare professionals in AI systems [52]. Regular audits of model predictions for demographic parity and equity metrics hold significance for responsible deployment in diverse clinical populations [53].

## 5. Conclusion

This single center study shows that the Random Forest algorithm enhanced by RFE feature selection and functional evaluation has high accuracy in predicting exercise intolerance in maintenance hemodialysis patients during dialysis. The model results showed that functional reserve (represented by TUG testing) and hemodynamic baseline (pre dialysis SBP) were the dominant predictors of exercise intolerance, with higher importance than static demographic factors. Through sex subgroup validation and SHAP analysis to enhance model interpretability, this study provides a potential predictive tool for precision nephrology. However, due to the single center design and lack of external validation in this study, the generalization ability of the model still needs further confirmation through multi center and large sample studies. Based on the research results, we suggest considering routine inclusion of functional exercise tests in dialysis clinical practice, not only for rehabilitation monitoring, but also as an important reference indicator for risk stratification. Future research needs to optimize the model through external validation and explore its applicability in different dialysis populations.

## Supplementary Material

Supplementary.docx

## Data Availability

The data presented in this study are available on request from the corresponding author.
