# Supplementary material for: Predicting intradialytic exercise intolerance in maintenance hemodialysis patients: an interpretable machine learning approach integrating functional assessments
Source: Ren Fail. 2026 Jun 23;48(1):2689781. doi: 10.1080/0886022X.2026.2689781 (PMC13292310; doi:10.1080/0886022X.2026.2689781)
Supplement: Supplementary.docx [file IRNF_A_2689781_SM7051.docx]

Table S1. Sensitivity analysis comparing model performance between the Original Feature Set and Derived Feature Set.

| Feature Set Configuration | ROC AUC (Mean ± SD) | Accuracy (Mean ± SD) | F1 Score (Mean ± SD) | Brier Score (Mean ± SD) |
| --- | --- | --- | --- | --- |
| Original Set | 0.914 ± 0.024 | 0.845 ± 0.025 | 0.750 ± 0.047 | 0.120 ± 0.010 |
| Derived Set | 0.906 ± 0.029 | 0.831 ± 0.032 | 0.738 ± 0.055 | 0.135 ± 0.024 |

Table S2. Feature ranking and selection results based on RFE.

| Feature Name | Category | RFE Ranking | Status |
| --- | --- | --- | --- |
| age_years | Demographics | 1 | Selected |
| TUG_sec | Functional Test | 1 | Selected |
| pre_SBP_mmHg | Hemodynamics | 1 | Selected |
| planned_UF_rate_mlkg_h | Dialysis | 1 | Selected |
| pre_Borg_RPE_0_10 | Functional Test | 1 | Selected |
| grip_trial1_kg | Functional Test | 1 | Selected |
| STS30_reps | Functional Test | 1 | Selected |
| dialysate_temperature_C | Dialysis | 1 | Selected |
| planned_total_UF_ml | Dialysis | 1 | Selected |
| gait_speed_4m_mps | Functional Test | 1 | Selected |
| pre_HR_bpm | Hemodynamics | 1 | Selected |
| pre_DBP_mmHg | Hemodynamics | 1 | Selected |
| pre_weight_kg | Anthropometry | 1 | Selected |
| height_cm | Anthropometry | 1 | Selected |
| dialysis_vintage_months | Dialysis | 1 | Selected |
| exercise_plan_intensity_cat | Exercise Plan | >1 | Eliminated |
| antihypertensive_taken_today | Medication | >1 | Eliminated |
| vascular_access_type | Dialysis | >1 | Eliminated |
| sex (Male/Female) | Demographics | >1 | Eliminated |

Table S3. Final hyperparameters of each model

| Model | Hyperparameter | Final Optimized Value |
| --- | --- | --- |
| Random Forest | n_estimators (Number of trees) | 500 |
|  | max_depth | 20 |
|  | min_samples_split | 10 |
|  | class_weight | Balanced |
| HGBDT | learning_rate | 0.1 |
|  | max_iter (Boosting iterations) | 300 |
|  | l2_regularization | 0.5 |
| XGBoost | learning_rate | 0.1 |
|  | n_estimators | 500 |
|  | max_depth | 6 |
| Logistic Regression | C (Inverse regularization strength) | 10.0 |
|  | l1_ratio (ElasticNet mixing) | 0.5 |
|  | Solver | SAGA |

The outcome of exercise intolerance exhibited a weak clustering effect (ICC=0.23, 95% CI: 0.17~0.29, p<0.001), indicating a correlation among multiple dialysis records for the same patient, but the degree of clustering is controllable. There was no significant difference in predictive performance between the multilevel mixed-effects model and the original Random Forest model (AUC: 0.912 vs. 0.914, p=0.82), confirming that the 10-fold GroupKFold cross-validation had effectively controlled for repeated measurement bias, and the results of the original model were reliable.

Table S4. Comparison of Performance Between Multilevel Mixed-Effects Model and Random Forest Model

| Metric | Mixed-Effects Model | Random Forest Model | p-value |
| --- | --- | --- | --- |
| ROC AUC | 0.912±0.026 | 0.914±0.024 | 0.82 |
| Accuracy | 0.841±0.028 | 0.845±0.025 | 0.76 |
| F1 Score | 0.746±0.049 | 0.750±0.047 | 0.81 |

Table S5. Comprehensive performance comparison of four prediction models across subgroups.

| Model | Subgroup | ROC AUC | Accuracy | F1 Score |
| --- | --- | --- | --- | --- |
| Random Forest | Overall | 0.914 ± 0.024 | 0.845 ± 0.025 | 0.750 ± 0.047 |
|  | Male | 0.919 ± 0.020 | 0.850 ± 0.029 | 0.737 ± 0.055 |
|  | Female | 0.905 ± 0.054 | 0.835 ± 0.051 | 0.764 ± 0.083 |
| HGBDT | Overall | 0.891 ± 0.028 | 0.834 ± 0.029 | 0.750 ± 0.059 |
|  | Male | 0.896 ± 0.035 | 0.837 ± 0.037 | 0.736 ± 0.072 |
|  | Female | 0.888 ± 0.066 | 0.832 ± 0.071 | 0.774 ± 0.094 |
| XGBoost | Overall | 0.891 ± 0.042 | 0.827 ± 0.036 | 0.744 ± 0.052 |
|  | Male | 0.900 ± 0.038 | 0.835 ± 0.039 | 0.742 ± 0.061 |
|  | Female | 0.875 ± 0.083 | 0.816 ± 0.067 | 0.748 ± 0.093 |
| Logistic Regression | Overall | 0.690 ± 0.071 | 0.628 ± 0.053 | 0.554 ± 0.078 |
|  | Male | 0.703 ± 0.085 | 0.651 ± 0.062 | 0.544 ± 0.101 |
|  | Female | 0.651 ± 0.116 | 0.578 ± 0.105 | 0.551 ± 0.130 |
|  |  |  |  |  |

Note: Values are presented as mean ± standard deviation calculated from the 10-fold cross-validation. The Random Forest model demonstrated superior performance overall and maintained robust predictive capability across both sex subgroups.


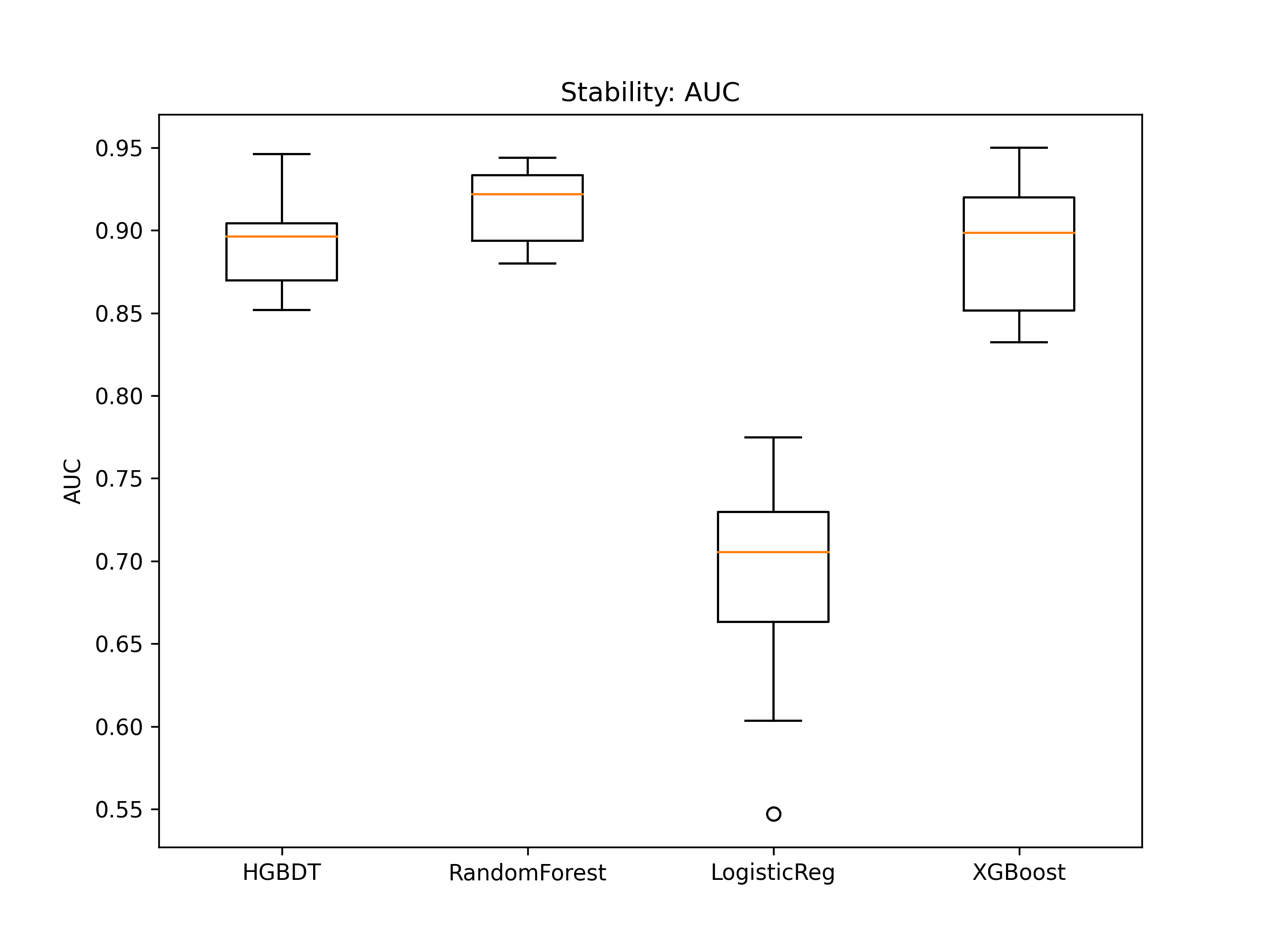


Figure S1. ROC AUC distribution.


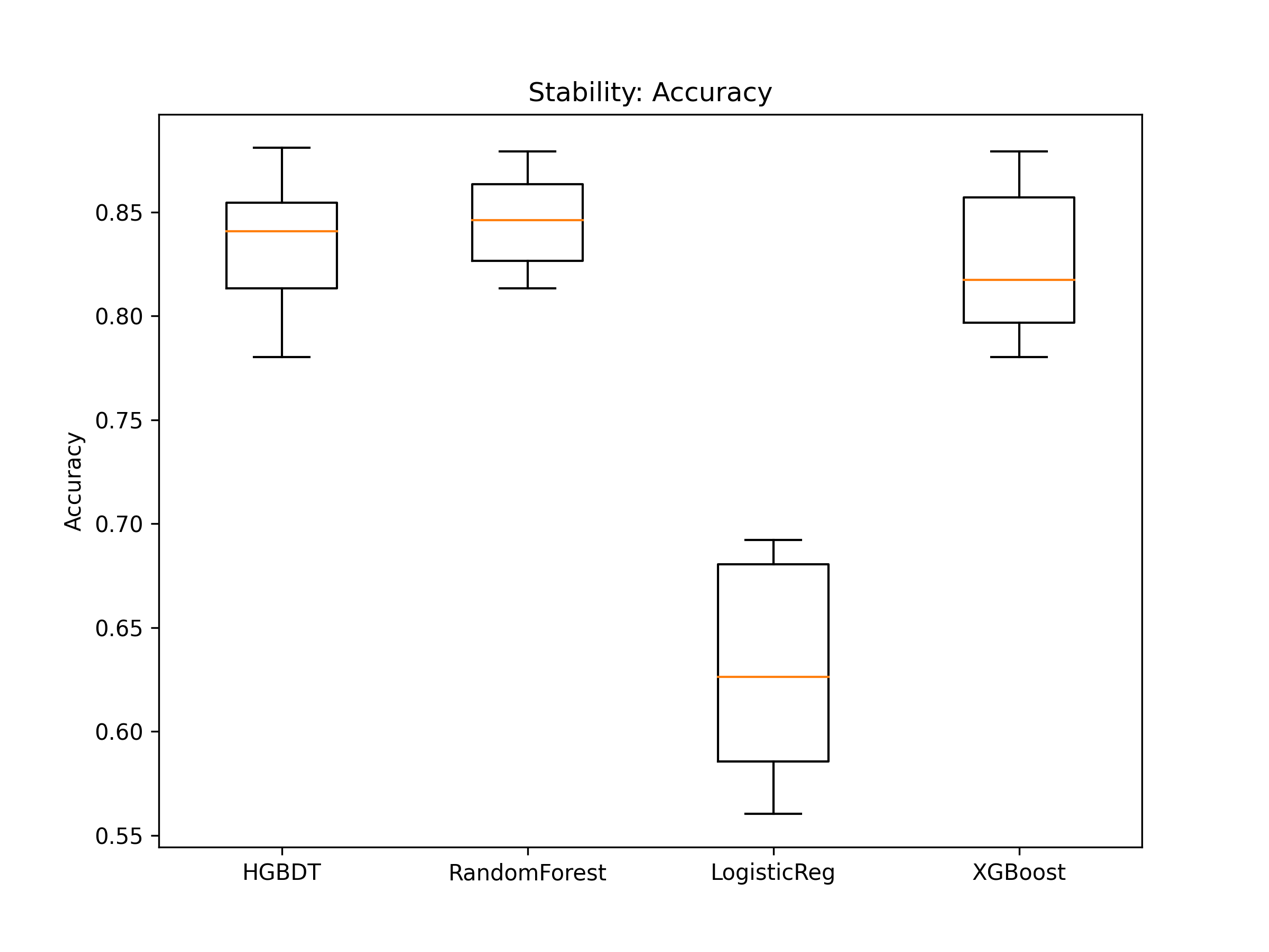


Figure S2. Accuracy distribution.


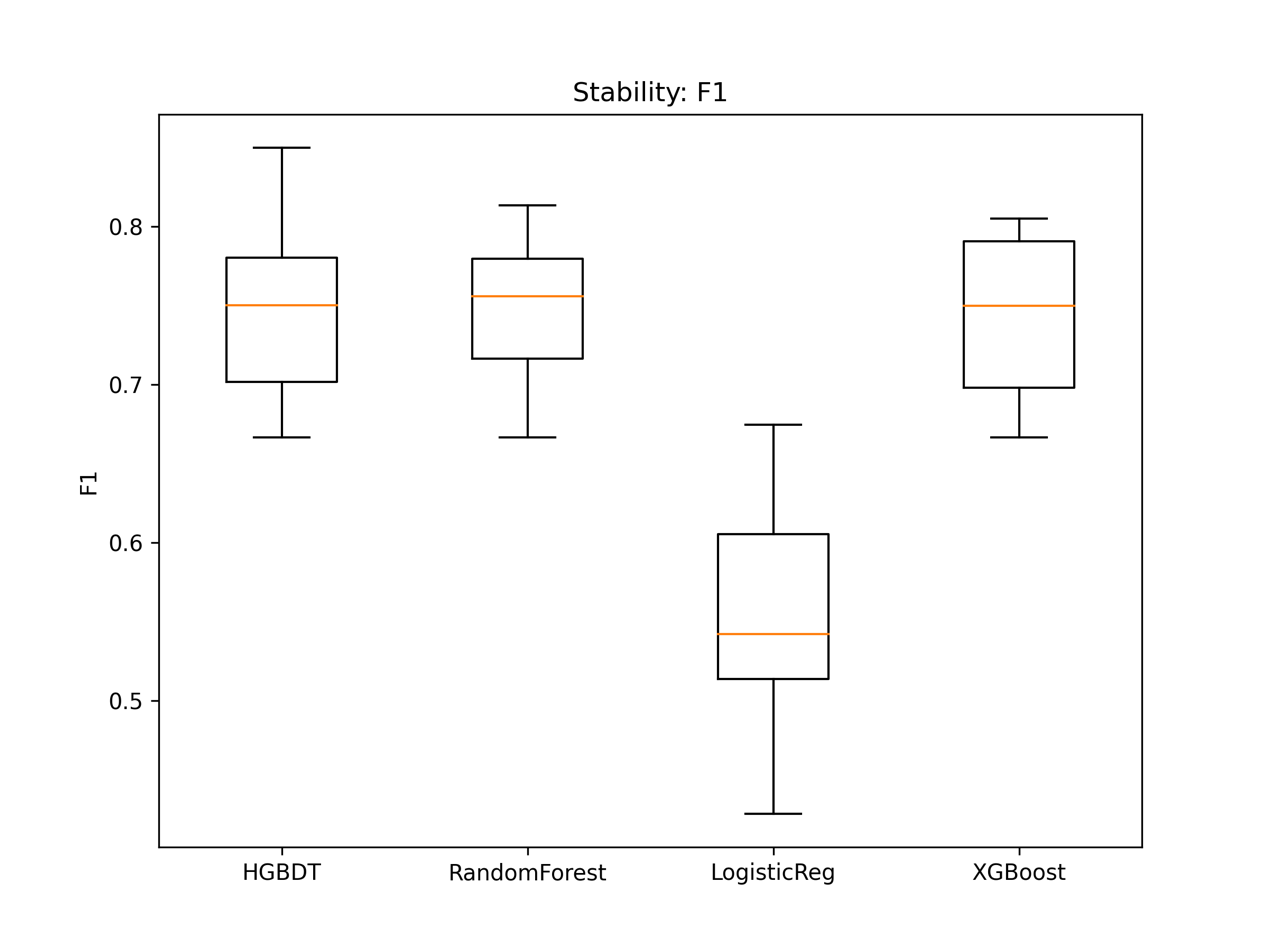


Figure S3. F1 Score distribution.
